# Years of Life Lost (YLLs) Due to Suicide and Homicide in Ilam Province: Iran, 2014-2018

**DOI:** 10.30476/BEAT.2022.92045.1293

**Published:** 2022-01

**Authors:** Yousef Veisani, Amin Bakhtiyari, Fathola Mohamadian

**Affiliations:** 1 *Psychosocial Injuries Research Center, Ilam University of Medical Sciences, Ilam, Iran*; 2 *Legal Medicine Research Center, Legal Medicine Organization, Tehran, Iran*; 3 *Department of Psychology, Psychosocial Injuries Research Center, Ilam University of Medical Sciences, Ilam, Iran*

**Keywords:** Suicide, Homicide, Years of life Lost, YLLs

## Abstract

**Objective:**

To provide detailed of suicide and homicide mortality and calculate of years of life lost (YLLs) in Ilam province Iran, during 2014-2018.

**Methods:**

In this cross-sectional study, all deaths due to suicide and homicide were enrolled to estimate YLLs, in Ilam province between 2014-2018. The source of data was legal medicine organization (LMO). All analysis was performed at 0.05 significant levels using statistical software package STATA for Windows version 11.2 and SPSS 21 software.

**Results:**

The total YLLs of suicide and homicide were 15685 and 5317, respectively. 522 per 100,000 populations were suicide and 117 for homicide. The YLL and 95% confidence interval form suicide was 34.4 (32.8-36.1) for both sexes that 33.7 (31.6-35.8) for men, and 35.5 (32.7-38.3) for women. In this study period, YLLs rate began to increase over the years in both injury-related in 2016.

**Conclusion:**

Results of this study disclosed the most prominent contribution of men and peoples aged 15-29 to the YLLs. Also our results indicate a recent increase in suicide and homicide YLLs for both genders.

## Introduction

Suicide and homicide have been a major causes of premature death worldwide and they cause social and economic burden on society [1]. In 2018, 1.4% of all deaths occurred due to suicide. Suicide is the 2^nd^ leading cause of death for peoples aged 15-29 and homicide ranked 3^rd^ for the same age group [2]. Suicide burden is gradually projected to increase from 1.8% in 1998 to 2.4% in 2020 [3]. In addition to suicide, homicide also has alarming statistics, therefore, 470,000 homicides have recorded in the world annually. About half of them occur in the 10-29 years of age group and 84% of homicides occur in men [4].

In Iran, suicide rate was 4.3 per 100,000 populations and 4% of total injuries are suicide. Homicide rate was occurred on 6.5 per 100,000 that 80% of victims were men and the mean age of victims were 32.4 year [5].

In Ilam, suicide has increased gradually in the last decade [6]. In Ilam province, the suicide rate was 19.5 per 100,000 people in 2014-2018 [7]. The national study showed that Ilam has a leading rank of suicide rate among 31 provinces in Iran, 2010 [8]. Based on recent national data, Ilam rates are higher than the national rates for both homicides and suicides. Ilam’s homicide rate is 8.1 per 100,000 while the national rate is 6.1 per 100,000. Ilam’s suicide rate is 20.7 per 100,000 while the national suicide rate is 5.1 per 100,000 [9]. The highest suicide attempts were observed in peoples aged 15-24. The overall suicide and homicide rate in aged 15-24 were 33.9, and 9.1 per 100,000, respectively [10]. 

The years of life lost (YLLs) was a versatile index to estimate the burden of injury-related mortality. YLLs have been widely used to evaluate disease burden in public health [11]. In Iran, YLLs rate due to suicide is similar with European countries, although the suicide mortality is much lower, young people are more likely to commit suicide in Iran [12].

Data collection and analysis is essential to understand the problem and support the government and public health agencies for designing intervention strategies and identify risk and protective factors. Therefore, the purpose of this study is to provide detailed of suicide and homicide mortality and calculate of YLLs in Ilam province Iran, during 2014-2018. 

## Materials and Methods

The present study is a cross-sectional on population living in Ilam province between 2014-2018. The source of data was legal medicine organization (LMO) in Ilam province and all deaths due to suicide and homicide were enrolled in the study. In line with the national laws in Iran, all violent death should be referred to LMO and death certificate is just issued by LMO. 

Data were extracted from medical records and death certificate in LMO. The variables in the study were age, sex, marital status, level of education, place of residence, cause of death, years of life lost due to premature death and life expectancy.

*Statistical Analysis*

The YLLs rats was calculated by decreasing the age of death according to standard life expectancy at a specific age and sex group, through the formula YLL=N*L which in formula N is the total number of deaths (in a specific age and sex group) and L is the life expectancy in the same age and sex group. A Chi-square test was performed to observe the significant differences between groups. All analysis was performed at 0.05 significant levels using statistical software package STATA for Windows version 11.2, and SPSS 21 software.

## Results

We combined data in five years 2014-2018 for YLL estimation in suicide and homicide. The total numbers of suicide in the study period were 445 (75.8). The highest rate of suicide was observed 272 (59.8) in men (*p*<0.003), and 15-29 age groups years 206 (45.3) (*p*<0.001), and city residence) (*p*<0.001). The YLL and 95% confidence interval form suicide was 34.4 (32.8-36.1) for both sexes; 33.7 (31.6–35.8) for men and 35.5 (32.7–38.3) for women. Suicide YLL for single and married peoples were 46.6 (45.8–47.4) and 21.3 (19.0–23.7), respectively. Also suicide YLL based on educational level illiterate/ primary school guidance/high school, diploma and academic degree was 27.6 (24.3-30.8), 41.0 (41.8-43.5), 38.4 (38.5-39.5) and, 38.7 (35.9-41.4), respectively. Suicide YLL were 35.1 (33.3-36.9) and, 30.1 (25.1-35.1) in cities and village residence, respectively (Table 1). 

**Table 1:** The demographic covavarits data of YLLs 95% CI and age standardized YLL rates for suicide of Ilam province, 2014-2018

		**Suicide**			
		**N (%)**	*p* **-value**	**YLL (95%CI)**	**ASR** ^a^
Gender	Male	272 (59.8)	0.003	33.7 (31.6-35.8)	1597
	Female	183 (40.2)		35.5 (32.7-38.3)	1017
Marital status	Single	236 (51.9)	0.389	46.6 (45.8-47.0)	1386
	Married	219 (48.1)		21.3 (19.0-23.7)	1228
Educational Status	Illiterate/ Primary school	202 (44.4)	<0.001	27.6 (24.3-30.8)	1186
	Guidance/high school	145 (31.9)		41.0 (41.8 -43.5)	851
	Diploma	92 (20.2)		38.4 (38.5-39.5)	540
	Academic degree	14 (3.3)		38.7 (35.9-41.4)	88
Residence	City	394 (86.6)	<0.001	35.1 (33.3-36.9)	2314
	Village	61 (13.4)		30.1 (25.1-35.1)	300
Total		445 (100)		34.4 (32.8-36.1)	2614

^a^Age Standardized YLL Rates per 100,000 Population

The total numbers of homicide in the study period were 145 (24.2). Also homicide more observed 116 (80%) in men (*p*<0.001), single persons 88 (60.7%) (*p*<0.006), 15-29 age groups years 68 (46.9%) (*p*<0.001), and city residence 18 (12.4%) (*p*<0.001). The YLL and 95% confidence interval form homicide was 36.9 (34.4-39.4) for both sexes, 36.9 (34.3-39.5) for men, and 36.9 (29.7-44.1) for women (Table 2). Homicide YLL for single and married persons were 45.4 (43.9-46.9) and 23.8 (19.9-27.8), respectively. Also, homicide YLL based on educational level illiterate/ primary school guidance/high school, diploma and, academic degree was 34.07 (28.4-39-6), 38.1 (35.3-40.6), 40.3 (37.4-43.1) and, 40.2 (38.1-42.3), respectively. Homicide YLL in cities and village residence were 37.3 (34.7-39.9) and, 34.1 (25.5-42.6), respectively (Table 2).

**Table 2:** The demographic data covavarits of YLLs 95% CI and age standardized YLL rates for homicide of the Ilam province, 2014-2018

		**Homicide**			
		**N (%)**	***p*** **-value**	**YLL (95%CI)**	**ASR** ^a^
Gender	Male	116 (80.0)	<0.001	36.9 (34.3-39.5)	707
	Female	29 (20.0)		36.9 (29.7-44.1)	179
Marital status	Single	88 (60.7)	0.006	45.4 (43.9-46.9)	535
	Married	57 (39.3)		23.8 (19.9-27.8)	351
Educational Status	Illiterate/ Primary school	58 (40.0)	<0.001	34.07 (28.4-39-6)	350
	Guidance/high school	58 (40.0)		38.1 (35.3-40.6)	350
	Diploma	24 (16.6)		40.3 (37.4-43.1)	147
	Academic degree	57 (2.8)		40.2 (38.1-42.3)	24
Residence	City	127 (87.6)	<0.001	37.3 (34.7-39.9)	775
	Village	18 (12.4)		34.1 (25.5-42.6)	111
Total		144 (100)		36.9 (34.4-39.4)	886

^a^Age Standardized YLL Rates per 100,000 Population

The total YLLs of suicide were 15685 (2614 per 100,000 populations) and 5317 YLLs results in in homicide (886 per 100,000 populations) (Table 3).

**Table 3:** The counts of YLL and age standardized YLL rates for suicide and homicide of the Ilam province by the year, 2014-2018

**Year**	**Suicide**			**Homicide**		
	**Counts**	**YLL**	**ASR (YLL)** ^a^	**Counts**	**YLL**	**ASR (YLL)** ^a^
2014	113	3822	637	39	1525	254
2015	88	3250	541	20	613	102
2016	76	2502	417	23	830	138
2017	81	2649	441	42	1579	263
2018	97	2013	335	20	769	128
Total	455	15685	2614	144	5316	886

^a^Age Standardized YLL Rates per 100,000 Population

**Fig. 1:**
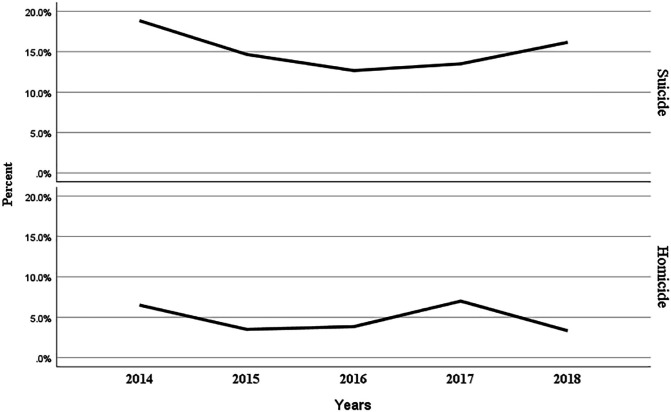
The annual percent change of YLL for suicide and homicide, Ilam province, 2014-2018

The percentage of YLL rates over the years by outcome are shown in Figure 1. The year with the highest YLL rate among study period for both outcomes are final study year 2018.

## Discussion

This study has been produced to provide the suicide and homicide mortality and calculate the YLLs in total. By main covariates, YLLs rate showed that the total YLL in suicide was 34.4 years and men had a higher contribution to YLL over the period of the study. Brazinova in own study revealed that the suicide risk is higher in men than in women [13]. Results of this study showed that the largest YLLs burden (45.3%) belonged to people aged 15-29 years in both genders. In other study in Iran, the annual self-harm mortality rate was higher in people aged 15-24 years, as well as in men in over a period of 26 years [14]. Curtin *et al*., showed that suicide attempts are more common in individuals 15-29 years old [15]. Fond *et al*., reported higher suicide rates in men and individuals aged 15-29 in 25 European countries and United States of America [16]. Based on result of the Global Burden of Disease (GBD), 1.3% of total DALYs were related to self-harms and suicide rank was 3 during 1990-2016 [17]. The reason for this result is men and people aged 15-29 who are in more stressful situations than other groups. Also, they may have dissimilarities in attitudes toward suicide.

According to the results of this study, homicide was more occurred in men 116 (80%). A study was reported a 10 years’ trend of homicide and showed that Ilam province had a higher annually incidence in Iran (4.4 per 100,000 persons) [18]. Other studies that conducted in Europe and Asia countries reported much more prevalence in men [19, 20].

The total YLLs due to suicide and homicide were 522 and, 177 YLLs per 100,000 populations, in both genders, annually. A national study in 2020 showed that YLLs due to suicide was 345 per 100,000 populations [12]. 

We investigated YLLs rate over the years and resulted that suicide and homicide rates began to increase in 2016. The present study indicates that recent increases were for both suicide and homicide. A national study in Iran between 2006-2016 showed a significantly decreasing rate [9] whereas, the fairly constant slop in homicide incidence rate was reported in Russia between 2001-2009 [21]. Therefore, homicide have a different trend changes in different countries.

The results of the present study revealed a higher rate of YLLs in suicide and homicide in Ilam province compared to other region in Iran. Our results disclosed the most prominent contribution of male gender and persons aged 15-29 to the YLLs. Also our results indicate a recent increase in suicide and homicide YLLs for both genders. There is a national need to implement an effective health policy intervention in order to save the burden of suicide in Ilam by considering that most of such deaths are preventable.

## Declaration

### Ethics approval and consent to participate:

This study was undertaken with the approval of the ethical committee of Legal Medicine Organization, Islamic Republic of Iran (IR.LMO.REC.1398.042).

### Consent for publication:

The authors express their consent to the publication of the article.

### Conflict of interests:

Authors have no conflict of interest.

### Funding:

This study was supported by Legal Medicine Organization, in Ilam province.

### Authors’ contributions:

Yousef Veisani had participated in the study design, literature review, preparation, and editing of the manuscript. Amin Bakhtiyari had participated in the study design, data collection, preparation, and editing the manuscript. Fathola Mohamadian had participated in the study design, data collection, preparation, and editing of the manuscript. All authors reviewed the preliminary and final analyses, and the draft and final manuscripts. All authors read and approved the final manuscript.

### Acknowledgements:

We would like to thank health staffs of Legal Medicine Organization, in Ilam province, which help us in data collection.
